# Effect of A- or B-Site Sc Doping on Sintering Temperature, Crystal Structure, Microstructure, and Properties of BaZr_x_Ti_1−x_O_3_ Ceramics

**DOI:** 10.3390/ma16206635

**Published:** 2023-10-11

**Authors:** Kaituo Zhang, Tiantian Li, Yuan Xu

**Affiliations:** 1 Henan Key Laboratory of Wire and Cable Structures and Materials, School of Cable Engineering, Henan Institute of Technology, Xinxiang 453003, China; 2 Engineering Technology Education Center, Henan Institute of Technology, Xinxiang 453003, China; 3 Henan Institute of Technology, College of Materials Sciences and Engineering, Xinxiang 453003, China

**Keywords:** Sc doping, BaTiO_3_, ceramics, A-site or B-site substitution

## Abstract

BaZr_x_Ti_1−x_O_3_ (BZT) ceramics with different concentrations of Sc ions were prepared, and the effect of doping concentration on the crystal substitution type of BZT was studied. The substitution position of the Sc ion in BZT was related to its concentration. When the concentration of Sc ions was low (<1.0 mol %), it showed B-site substitution; otherwise, Sc ions showed A-site substitution. In addition, the effects of the Sc ion concentration on the sintering temperature, crystal structure, microstructure, and properties of BZT were also studied. The results showed that the introduction of Sc ions can reduce the sintering temperature to 1250 °C. When the concentration of Sc ions was 1.0 mol % and 2.0 mol %, the high dielectric constants of BZT were 14,273 and 12,747, respectively.

## 1. Introduction

In recent decades, new materials and technologies have developed rapidly, with many positive impacts in the fields of information, medicine, communications, and energy storage. Perovskite materials have attracted the attention and research of many researchers because of their properties and characteristics [1,2,3]. BaTiO_3_ is one of the most typical perovskite materials, and the material has excellent ferroelectric, piezoelectric, high dielectric constant, and nonlinear optical properties [4,5,6]. It is commonly used as a multilayer ceramic capacitor (MLCC). Generally, MLCC should have a high dielectric constant, low dielectric loss, high breakdown strength, etc.

As the temperature decreases to 120 °C (cubic to tetragonal C-T), 5 °C (tetragonal to orthorhombic T-O), and −90 °C (orthorhombic to rhombohedral), BaTiO_3_ undergoes three phase transitions (also known as the Curie temperature, Tc), and the process of the phase transition affect the properties of the material [7,8]. In order to improve the properties of BaTiO_3_ ceramics, metal ions are usually introduced during the preparation process, which is not only simple but also inexpensive. The doping of these ionic elements causes a shift in the Curie temperature and the broadening of a dielectric temperature spectrum, which provides an opportunity for MLCC dielectric materials to work in a wide range. Studies have shown that BaZr_x_Ti_1−x_O_3_ (BZT) can be formed by introducing Zr ions with larger ionic radius into BaTiO_3_, which can not only reduce the production of Ti^3+^ in BaTiO_3_ but also stabilize the chemical structure and reduce dielectric loss [9]. In general, with an increase in the Zr ion content of BaTiO_3_, the phase transition moves rapidly to a low temperature and shows the property of diffuse phase transition. When the Zr ion content is 15%, the three Tc values of BaTiO_3_ overlap with each other to form a dielectric peak and move to the low-temperature region. 

BZT ceramics typically require a high sintering temperature of around 1450 °C, which not only complicates the preparation process but also hinders efforts to reduce energy consumption. Research shows that the use of borate or glass sintering additives can reduce the sintering temperature to a great extent; however, some sintering additives may have adverse effects on the properties of ceramics. Studies have shown that the addition of metal oxides can improve the sintering state. Muhammad et al. reported that the use of Li_2_CO_3_ can reduce the sintering temperature of BaTiO_3_ from 1500 °C to 1350 °C [10]. Xu et al. introduced MgO into BaTiO_3_, which not only reduced the sintering temperature but also improved the properties of BaTiO_3_ [11]. In the sintering process of BaTiO_3_, the metal ions of some metal oxides can enter the lattice. Metal ions with larger ionic radius can easily replace A-site ions (the position of Ba ions in the BaTiO_3_ crystal), whereas smaller metal ions can easily replace B-site ions (the position of Ti ions in the BaTiO_3_ crystal) and enter the body-centered position of the oxygen octahedron [12,13]. When metal ions enter the lattice or interstitial void, the crystal produces different forms of distortion and destroys the long-range ordered state of the dipoles, resulting in changes in the Curie temperature, dielectric properties, and ferroelectric properties.

Recently, we reported the effect of a series of metal ions with different radii on the structure, microstructure, and properties of BZT [14]. Interestingly, we found that the ionic radius of Sc is 0.089 nm, which is between Ti ions (0.0605 nm), Zr ions (0.072 nm), and Ba ions (0.135 nm), and may replace A-site or B-site. In this work, we prepared BZT with different Sc ion doping concentrations using a solid-state reaction method.

Using X-ray diffraction (XRD), Raman spectroscopy, scanning electron microscopy (SEM), dielectric testing system, ferroelectric testing system, and density meter, the effects of Sc ions doping concentration on the sintering temperature, crystal structure, microstructure, dielectric properties, and ferroelectric properties of BaTiO_3_ were investigated.

## 2. Materials and Methods

In this work, we prepared BZT with Sc ion doping content of 0, 0.5, 1.0, 1.5, 2, and 2.5 mol % (BZT-x mol %) using a solid-state reaction method. In short, BaCO_3_, TiO_2_, ZrO_2,_ and Sc_2_O_3_ (Alfa Aesar) were weighed using a molar ratio, and an appropriate amount of ethanol was added to the nylon tank and ball mill for 24 h. Then, the powder material was sintered in a crucible at 1100 °C for 12 h. An appropriate amount of Sc_2_O_3_ was weighed and mixed with the sintered powder material, and the ceramic material was granulated using 5% polyethylene glycol (PVA). The granulated material was placed under a pressure of 15 Mpa for 10 min to obtain an embryonic preform sheet, and the embryonic preform sheet was fired in a furnace. Finally, both sides of the ceramic sheet were polished and the silver electrode was prepared.

The XRD of the SmartLab model produced by Rigaku Co., LTD., Tokyo, Japan, was used to test the crystal structures of the ceramics. The step size was 2°/min and the test angle was 20–80°. The micromorphology of the samples was observed via FR-SEM using a JSM-7610F Plus model manufactured by Nippon Electronics, Tokyo, Japan. The density meter was produced by Guangdong Hongtuo Instrument Technology Co., LTD. The dielectric spectrum was measured using a dielectric test system of Navocontrol technologies in Germany. The ferroelectric properties were measured using the Radiant Premier II ferroelectric test system produced in Germany, and the Raman spectra were measured using the inVi microconfocal Raman spectrometer produced by Renishaw in London, UK. 

## 3. Results and Discussion

### 3.1. Effect of Sc Ion Doping on Sintering Temperature

**Figure 1:**
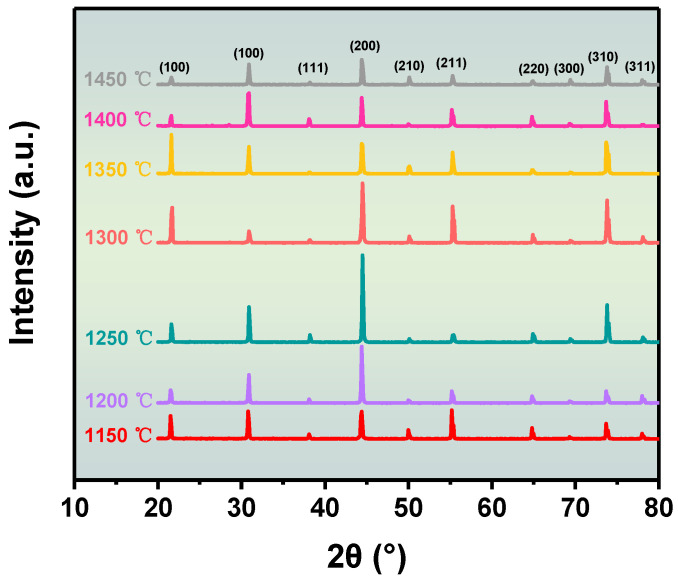
XRD patterns for the BZT-1.5 mol % ceramics at different sintering temperatures.

The BZT-1.5 mol % ceramics were selected as the research object, and the sintering temperature was controlled at seven temperatures of 1150, 1200, 1250, 1300, 1350, 1400, and 1450 °C for 2 h. Figure 1 and Figure S1 show the X-ray diffraction of BZT-1.5 mol % ceramics at different annealing temperatures. Figure 1 demonstrates that there are no stray peaks in all the diffraction peaks, indicating the successful preparation of the BZT-1.5 mol % ceramics and that the doping of Sc ions in BZT does not cause a phase change [15]. When the sintering temperature is 1150 °C, the XRD peak is weak. The X-ray diffraction peak of BZT was enhanced when the annealing temperature was increased, especially the (200) diffraction peak. When the sintering temperature rises to 1250 °C, the X-ray diffraction peak is the strongest. Therefore, we speculate that the crystallization of BZT is best when the sintering temperature is 1250 °C.

**Figure 2:**
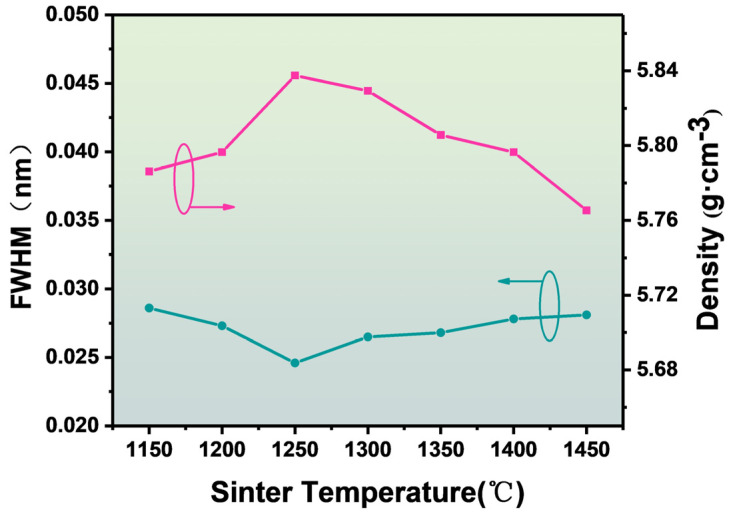
Density curve and FWHM curve of BZT-1.5 mol % ceramics at different sintering temperatures.

We calculated and fitted the full width at half maxima (FWHM) value of the XRD diffraction peak, as seen in Figure 2. The FWHM value (the data below the figure; the green line) gradually decreases with the increase in sintering temperature and has a minimum value of 1250 °C [16]. According to the Bragg diffraction equation, the value of the FWHM is directly related to the crystallinity, and a smaller FWHM means a stronger crystallinity [17,18]. Figure 2 shows the density curve of the BZT-1.5 mol % ceramics at different annealing temperatures (the data above the figure; the red line). The density of the ceramics is 7.81 g cm^−3^ when the annealing temperature is 1150 °C. When the annealing temperature is increased to 1250 °C, the density is 5.83 cm^−3^, and the maximum density is reached. Then, the ceramic density gradually decreased. In general, the density of ceramics is related to their quality and crystallinity, so the density curve shows that a sintering temperature of 1250 °C may be the best sintering temperature for BZT-1.5 mol % ceramics. 

To more directly observe the effect of sintering temperature on the microstructure of BZT ceramics, SEM microstructure images at 1100, 1200, 1250, and 1350 °C are presented, as shown in Figure S2a–d. To be able to clearly observe the microscopic morphology of the sample surface, they were lightly polished before the test. In Figure S2a, when the sintering temperature is 1100 °C, the grain size is small, and as the temperature increases, smaller grains merge and fuse. As shown in Figure S2b, it can be clearly seen that the energy provided by the lower temperature is not enough, resulting in uneven grain growth, and some smaller grains do not merge. When the temperature rises to 1250 °C, the grains are full, dense, and uniform, which is consistent with the density curve in Figure S2c. When the sintering temperature is further increased, smaller gaps appear between the crystals, especially at the grain boundary position, which results in a decrease in the density of the ceramic sample (Figure S2d).

**Figure 3:**
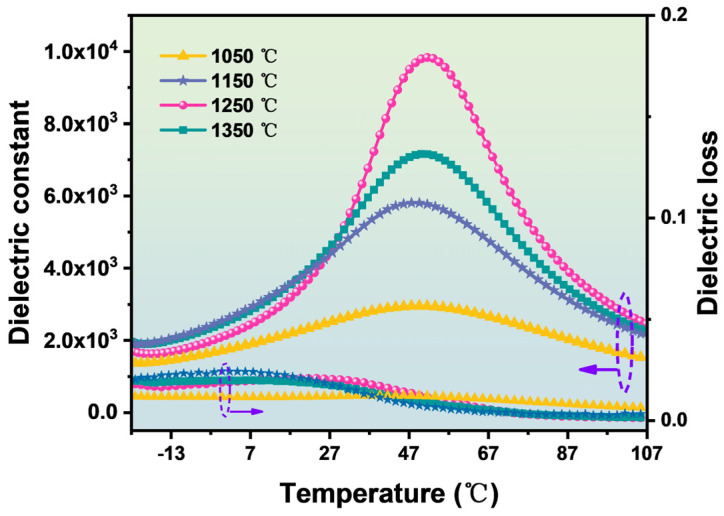
Curve of dielectric constant and dielectric loss of BZT−1.5 mol % ceramics with temperature.

In Figure 3, in order to further verify the optimal sintering temperature, the dielectric properties of the ceramic samples were also tested according to the dielectric temperature spectrum. In Figure 3, there is a strong correlation between the annealing temperature and dielectric constant of the BZT-1.5 mol % ceramics. The BZT ceramic samples with an annealing temperature of 1250 °C have the greatest dielectric constant, which means that they have the best sintering temperature. A suitable annealing temperature provides sufficient energy for grain boundary movement and grain merging. The number of grain boundaries per unit volume is small, which is conducive to increasing the dielectric constant and improving the dielectric properties [19,20,21]. At the same time, it can be clearly seen from the SEM photos, as shown in Figure S2a, that when the sintering temperature is 1100 °C, the grain size of BZT ceramics is small and there are more defects at the grain boundary, so it is not conducive to the improvement of the dielectric properties. As the sintering temperature increases, the defects generated by the crystal and the grain boundary gradually decrease, as shown in Figure S2c. When the annealing temperature is 1250 °C, the defects on the crystal surface and at the grain boundary position are minimal, so it has the best dielectric properties. In addition, due to the longer grain arrangement being more compact, the structure of the ceramics is also denser. Higher-density ceramic samples are conducive to improving the dielectric properties, which is consistent with the density curve in Figure 2. Therefore, when the sintering temperature is increased to 1250 °C, the energy provided by the external environment is sufficient, and this encourages sample crystallization and growth; this sample has the best dielectric properties. The energy provided by the external environment is sufficient and sufficient to crystallize and grow the sample, which means that it has the best dielectric properties [22,23]. 

If the temperature is too high, resulting in gaps or holes around the grain boundaries, it will cause abnormal grain growth or excessive grain growth; this is consistent with the density curve. The annealing temperature of BZT-1.5 mol % is mostly 1450 °C. Sc ions play a role as a sintering assistant, and their introduction is conducive to liquid-phase sintering in the sintering process of BZT, thereby reducing the optimal sintering temperature of BZT and saving energy.

### 3.2. Effect of Sc Ion Content on Crystal Structure of BZT Ceramics

**Figure 4:**
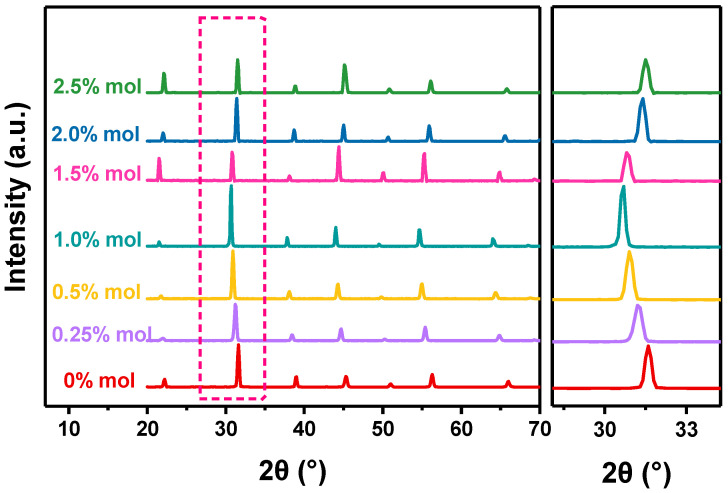
XRD patterns for the BZT−x mol % ceramics and enlarged regions of the XRD patterns.

We prepared BZT ceramics with Sc ion contents of 0, 0.5,1.0, 1.5, 2.0 and 2.5 mol %. Firstly, the effect of Sc ions on the crystal structure of BZT was analyzed. Figure 4 shows the XRD patterns of BZT-x mol % ceramics. It can be clearly seen that the introduction of Sc ions will not destroy the structures. From the fine pattern of XRD, the peak moves to a lower angle with the increase in Sc content, which means that the cell parameters become larger according to the Bragg diffraction equation. However, it is interesting that the XRD peak gradually develops a steeper angle as the Sc ion content continues to increase. This means that the cell parameters shrink as the Sc ion concentration exceeds 1%. The ionic radii of Ti^4+^, Zr^4+,^ and Ba^2+^ are 0.060, 0.072, and 0.161 nm, respectively, while the ionic radius of Sc ions is 0.089 nm. The ionic radius of Sc ions is more similar to the size of B-site ions (Ti ions and Zr ions), so the introduction of trace Sc ions to BZT is more prone to B-site substitution, resulting in lattice expansion. When the concentration of Sc ions exceeds 1.0 mol %, Sc ions gradually replace the A-site ions. As the ionic radius of an A-site ion is larger than that of a Sc ion, the resulting crystal lattice contraction and grain parameter values decrease [24].

**Figure 5:**
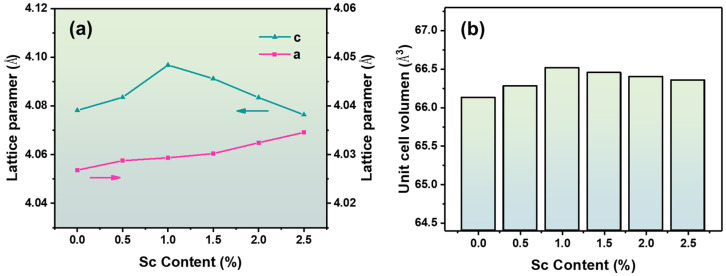
Crystal−related data for ceramics (**a**) lattice parameters and (**b**) cell volume.

In order to obtain the exact crystal lattice parameters of the sample, the GSAS-II program was used to carry out the structural refinement seen in Figure S4. The details of the Rietveld refinement results of BZT-x mol % ceramics are given in Table S1. The reliability value of the weighted patterns (R_wp_) is less than 10%, indicating the good fitting of the selected structural model. In Figure 5a,b, relevant data of the cell parameters with different Sc ion doping concentrations were obtained using a Rietveld refinement model. The A-axis of the cell is gradually stretched, and the C-axis is first stretched and then compressed. Figure 5b shows that when the content of Sc ions was less than 1.0 mol %, the cell volume gradually increased with the gradual increase in Sc ion content. The cell volume of the BZT-1.0 mol % ceramic sample reached the maximum, and subsequently, the cell volume gradually decreased with the increase in Sc ion content. Therefore, Sc ions are more inclined to replace the B position in BZT when the content of Sc ions is less than 1.0 mol %. Some Sc ions will replace the A-site of BZT when the concentration of Sc ions is greater than 1.0 mol %.

**Figure 6:**
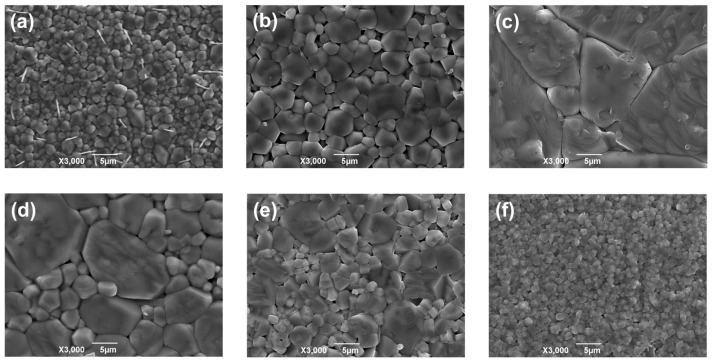
SEM images of BZT−x mol % ceramics (**a**) 0 mol %, (**b**) 0.5 mol %, (**c**) 1.0 mol %, (**d**) 1.5 mol %, (**e**) 2.0 mol %, and (**f**) 2.5 mol %.

In Figure 6a–f, to study the effect of Sc introduction on the microstructure of BZT. Figure 6a shows the SEM image of-BZT-0 mol %. In order to analyze the grain size, we measured and calculated the average grain size, and relevant information is shown in Figure S3. The grain size is not uniform and is generally small, and the average grain size is 1.32 μm. The void around the grain boundary obviously decreases in size, and the grain size becomes larger and uniform gradually when Sc ions are introduced. When the doping concentration of Sc ions is 0.5 mol %, the grain size becomes significantly larger, as shown in Figure 6b, and the average grain size is 3.05 μm. As shown in Figure 6c, when the Sc ion content is 1.0 mol %, the grain size reaches the maximum of 10.67 μm, the grains are full, the number of grain boundaries per unit volume is lower, and the overall structure is denser. Sc ion solids dissolved into the crystal structure in the sintering process (to play the role of sintering aid) reduce the sintering temperature point of ceramics so that grain growth occurs. Reducing the sintering temperature point of ceramics means increasing their sintering temperature. Similar conclusions can be drawn by controlling the sintering temperature to control the grain size [10]. With the increase in Sc ion content, grain growth is inhibited; Figure 6d shows that the average grain size is 4.15 μm. More Sc ions accumulate at the grain boundary to produce a pinning effect to inhibit grain growth; alternatively, this may be related to Sc ions replacing A-site ions in BZT [25,26]. As shown in Figure 6e,f, when the concentration of Sc ions is increased to 2.0 and 2.5 mol %, the average grain size is only 2.27 and 0.81 microns. With the increase in the doping amount of Sc_2_O_3_, Sc ions not only exist in the crystal structure but also may exist at the grain boundaries, thus inhibiting the growth of grains. We analyzed the element distribution of BZT ceramics via EDX, as shown in Figure S5 and Table S2. It shows that Sc ions are very evenly dispersed throughout the BZT ceramics, which is consistent with XRD results. In addition, when the concentration of Sc ions is greater than 1.0 mol %, Sc gradually performs B-site substitution. Since the radius of Sc ions is larger than those of Ti and Zr ions, the fluidity of Sc ions after entering the lattice is reduced, which is not conducive to the merging of small grains. Therefore, when the Sc ion content is greater than 1.0 mol %, the grain size decreases.

**Figure 7:**
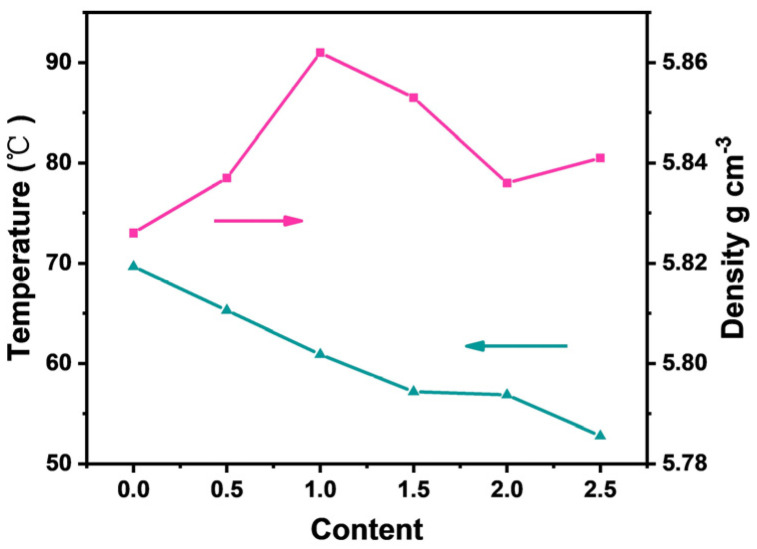
Curie temperature and Density curves of BZT−xmol % ceramics.

In Figure 7, we show the density curve of the BZT-x mol % samples measured using Archimedes’ principle. The density of BZT gradually increases with the increase in Sc content and the relative density is the highest when the Sc ion content is 1.0 mol %. In general, the density of ceramics is directly related to the properties of the material. For ceramic materials, a higher density means that pores and bubbles are less likely to appear inside the ceramics, which is beneficial to inhibiting the dielectric loss of the materials and improving their electrical properties, which is more consistent with the description of SEM. With the increase in Sc ion content, grains gradually filled the entire space, and the grains are full and dense, and the density reaches the maximum when the Sc ion content is 1.0 mol %, as shown in Figure 6c. When the Sc ion content exceeds 1.0 mol %, crystal growth is inhibited with the increase in Sc ion content, and there is a void in the position where the grain makes contact with another grain, resulting in a decrease in density. In addition, the density of ceramics is also related to the grain size, and the more grains per unit volume in the ceramic material there are, the more grain boundaries it produces. Grain boundaries are less dense than the grains. In the crystal crystallization process, some impurities or pores are pushed to the grain boundary, so the grain boundary is another form of defect, which is not conducive to the improvement of the ceramic properties.

**Figure 8:**
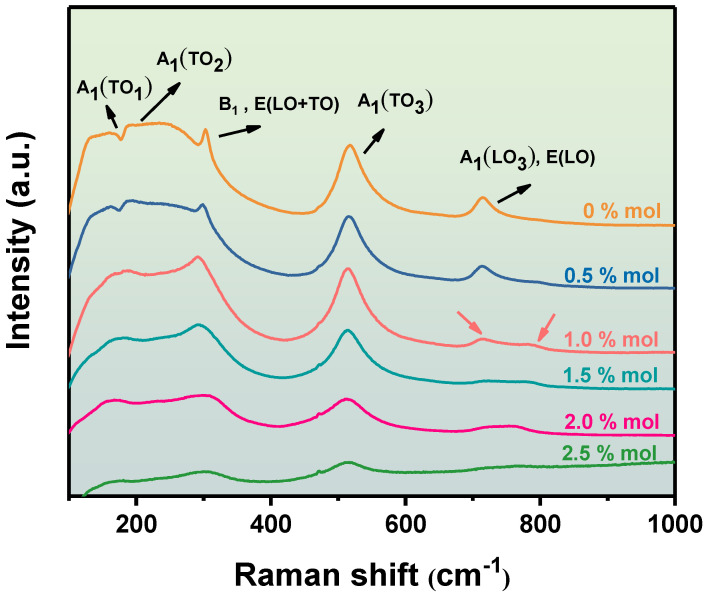
Raman spectra of the BZT−x mol % ceramics.

Figure 8 shows the Raman spectra of BZT ceramics doped with Sc ions at room temperature. In general, A_1_(TO)~182, A_1_(TO_2_)~265, A_1_(TO_3_)~521, and A_1_(LO_3_) and E(LO)~725 cm^−1^ represent the crystallization peaks of BZT ceramics, which are consistent with previous reports [14,27]. The A_1_(TO_1_) peak near 182 cm^−1^ is mainly caused by Ba ions in the A position. The broadening or weakening of the vibration peak is mainly due to the polycrystalline nature of perovskite ceramics and the static disorder caused by the random substitution of Sc ions with Ba ions [28,29]. It can be clearly seen from Figure 8 that with the increase in Sc ion content, the intensity of the A_1_(TO_1_) vibration peak gradually decreases and widens, especially when the Sc ion content is 1.0 mol %, which is consistent with the above analysis. When the content of Sc ion was more than 1.0 mol %, Sc ions gradually replaced the A-site ions. The peaks at 295–310 cm^−1^ are usually associated with the cubic phase and the tetragonal phase of BZT ceramics [30,31,32]. As shown in Figure 8, the peak is gradually broadened and weakened with the increase in Sc ion content, which means that the ceramic gradually changes from the tetranuclear phase to the cubic phase. When the Sc ion content is greater than 1.0 mol %, the ceramics have larger lattice parameters, which is consistent with the above analysis of lattice parameters. The A_1_(TO_3_) peak near 521 cm^−1^ is mainly related to the ferroelectric phase transition and the long-range order of dipoles [33,34]. With the increase in Sc ion content, the intensity of A_1_(TO_3_) gradually weakens, indicating that the BZT ceramics gradually transition to a relaxed ferroelectric. The Raman peaks of A_1_(LO_3_) and E(LO) are generally related to the oxygen octahedral structure of BZT ceramics [35,36]. The peaks of A_1_(LO_3_) and E(LO) adopt a high wave number position with the increase in Sc ion content. There is a small peak near 795 cm^−1^ of BZT-1.0 mol %, which means that Sc ions gradually replace the A-site ions of BZT ceramics. The small peak gradually moves to a low wave number position with the increase in Sc ion content. A small peak is merged with A_1_(LO_3_) and E(LO) when the Sc ion content is 2 mol %, which means that Sc ions replace A-site ions more.

### 3.3. Effect of Sc Ion Content on Dielectric Properties of BZT Ceramics

**Figure 9:**
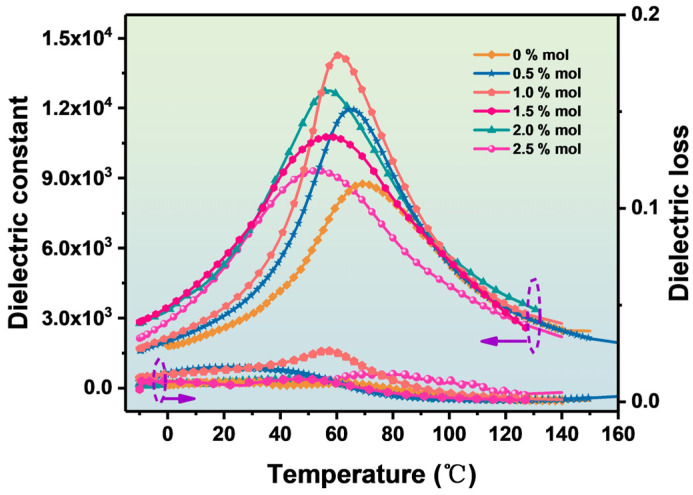
Curve of dielectric constant and dielectric loss of BZT−x mol % ceramics with temperature.

Figure 9 shows the dielectric temperature spectra with different contents of Sc ions measured at 1 kHz. From Figure 9, it can be seen that the dielectric properties of the BZT ceramics are obviously improved after Sc ions are introduced into the BZT ceramics. The permittivities of the BZT ceramic samples with 1.0% Sc ions and 2.0 mol % are 14,273 and 12,747 at the Curie temperature, respectively. The BZT-1.0 mol % ceramic samples have the largest and most uniform grain size, which is conducive to improving the density of ceramics and the dielectric properties. Smaller and non-uniform grains produce more grain boundaries per unit volume, and grain boundaries inhibit dipole inversion and dielectric enhancement [25]. In addition, the proper introduction of Sc ions is beneficial to improve the crystal structure and ferroelectric phase state of BZT ceramic crystals, which has been explained in Raman spectral analysis. When excessive Sc ions are introduced into BZT ceramics, they not only replace the B-position ions of BZT ceramic crystals but also replace the A-position ions. Sc ions replacing A or B ions of BZT ceramic crystals will produce a large number of receptor vacancies, and more receptor vacancies can improve the dielectric properties of BZT ceramics. Therefore, the dielectric constant of BZT-2.0 mol % ceramics can reach 12,747, as shown in Figure 9 [22]. To analyze the influence of Sc ions on the Curie temperature properties, the correlation curves of BZT ceramics with different concentrations of Sc ions are shown in Figure 7. The Curie temperature can be adjusted after the introduction of Sc ions. The Curie temperature becomes low with the increase in Sc content, and the Curie temperature approaches room temperature when the Sc ion content is 2.5 mol %. This is related to the structure of oxygen octahedron BZT ceramics and the change in grain size.

**Figure 10:**
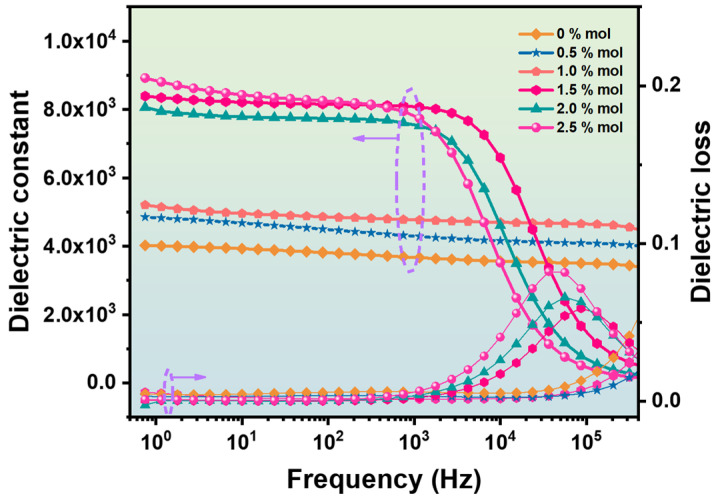
Curve of dielectric constant and dielectric loss of BZT−x mol % ceramics with frequency.

In order to further analyze the dielectric properties of BZT ceramics, Figure 10 shows the dielectric spectrum curves of BZT ceramic samples with different Sc ion contents tested at room temperature. The BZT ceramic samples with Sc ion contents of 1.5, 2.0, and BZT-2.5 mol % have a large dielectric constant at the lower frequency portion of the permittivity curve. The permittivity of BZT ceramics with a Sc ion content less than 1.5 mol % changes minimally with frequency, while that of the samples with a Sc ion content greater than 1.5 mol % changes greatly with frequency; in particular, there is a steep drop in the high-frequency region. In general, the low-frequency region mainly reflects interface polarization and molecular displacement polarization, which corresponds to the vacancies, bubbles, and interlayers in the ceramic sample [37]. When the content of Sc ions exceeds 1.0 mol %, Sc ions act as acceptors in BZT ceramics and produce a large number of vacancies. In addition, an excessive Sc ion content also inhibits grain growth, resulting in more grain boundaries. Therefore, BZT 1.5, 2.0, and 2.5 mol % ceramic samples have a larger permittivity in the lower frequency portion. The most important role in the higher frequency portion, usually at the microscopic level, is adopted by processes such as ion displacement polarization and electron displacement polarization. For samples with a lower Sc ion content, the fluctuation is still small due to the intact crystal structure and there being fewer vacancies. For BZT ceramic samples with more Sc ions, more donor ions will produce more vacancies and grain boundaries, which will affect ion displacement polarization and electron displacement polarization in the higher frequency portion, and this will show large fluctuations in the dielectric spectrum [38].

**Figure 11:**
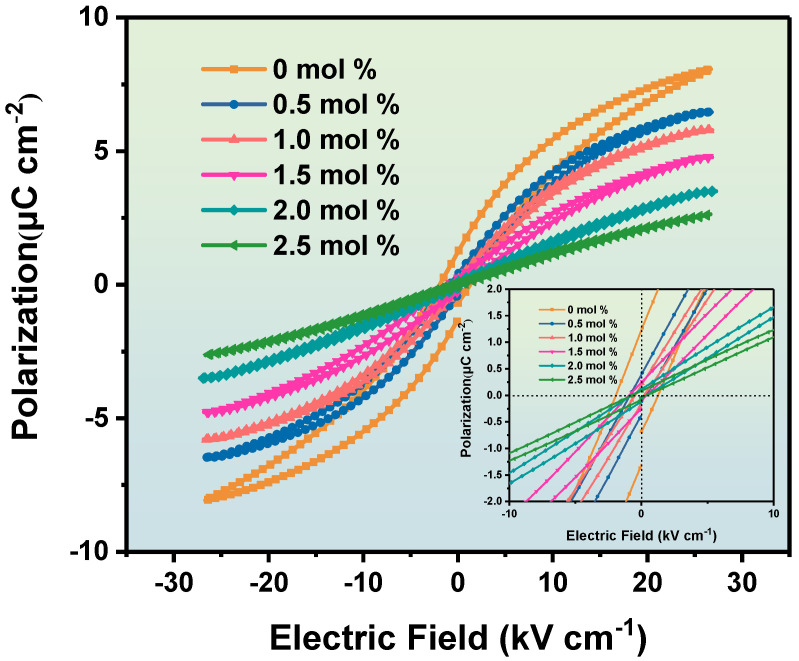
The hysteresis loops of the BZT−x mol % ceramics.

Figure 11 shows the hysteresis loops of BZT-xmol % ceramics measured at 1 kHz at room temperature. The area enclosed by the hysteresis loop of all the samples is thin, and this means that the Sc-doped BZT ceramics are relaxed ferroelectrics [39]. In general, ferroelectric properties are related to the crystallinity, grain size, lattice defects, and domain structure of ceramics [40]. The crystal structure of the BZT ceramics gradually changes from a tetragonal phase to a cubic phase structure, which will lead to the weakening of the ferroelectric property with the increase in Sc ion content. It can be clearly seen from the illustration in Figure 11 that Pr is only 0.42 μC/cm^2^ when Sc ion content is 0.5 mol %. With the increase in Sc ion content, the Pr decreases to 0.1 μC/cm^2^ when Sc ion content is 2.5 mol %. The introduction of Sc ions into BZT ceramics destroys the long-range order of the octahedral structure of TiO6 in the BZT ceramic structure and induces the polarity nanoregion, which leads to the reduction in the residual polarization intensity Pr.

## 4. Conclusions

In this work, we investigated the substitution types of BZT crystals with different Sc ion concentrations and explored the effects of Sc ions on the annealing temperature, microstructure, crystal structure, and properties. The introduction of Sc ions can reduce the sintering temperature to 1250 °C and improve the sintering state of BZT ceramics. Sc ions can enter the crystal structure when their content is less than 1.0 mol %. It is more likely that they will replace the B position of BZT ceramics and gradually replace the A position with an increase in Sc ion content. When the content of Sc ions is 1.0 mol %, the BZT ceramics have larger and more symmetrical grain sizes and denser structures. The introduction of Sc ions can improve the dielectric properties of BZT ceramics. The dielectric properties of BZT ceramics are better when the contents of Sc ions are 1.0 and 2.0 mol %, and the mechanism of action is different. The Curie temperature moves to the low-temperature region with an increase in Sc ion content, and the samples show the characteristics of relaxed ferroelectrics. In addition, with an increase in Sc ion content, the residual polarization intensity decreases.

## Data Availability

Data are available from the corresponding author upon reasonable request.

## Supplementary Materials

The following supporting information can be downloaded at: https://www.mdpi.com/article/10.3390/ma16206635/s1, Figure S1. XRD patterns for the BZT-1.5mol % ceramics at different sintering temperatures. Figure S2. SEM images of BZT-1.5 % mol ceramics at sintering temperature of (a) 1150, (b) 1200, (c) 1250 and (d) 1350 °C. Figure S3. SEM images of BZT-x % mol ceramics (a) 0% mol, (b) 0.5% mol, (c) 1.0% mol, (d) 1.5 % mol, (e) 2.0% mol and (f) 2.5 mol and statistical chart. Figure S4. Rietveld refined room temperature XRD patterns for the BZT-x % mol ceramics. (a) 0% mol, (b) 0.25% mol, (c) 0.5% mol, (d) 1.0% mol, (e) 1.5 % mol, (f) 2.0% mol and (g) 2.5 mol. Figure S5. EDX map of 2.0% mol-BZT ceramics and correlation test parameter. Table S1. Lattice constants fitting precision parameters of x-mol-BZT ceramics refined by Rietveld method. Table S2. The content of elements is determined by EDX test.
