# Supplementary material for: Effect of A- or B-Site Sc Doping on Sintering Temperature, Crystal Structure, Microstructure, and Properties of BaZrxTi1−xO3 Ceramics
Source: Materials (Basel). 2023 Oct 11;16(20):6635. doi: 10.3390/ma16206635 (PMC10608409; doi:10.3390/ma16206635)
Supplement: Supplementary file 1 [file materials-16-06635-s001.zip › materials-2545444-Supplementary.pdf]

materials

Supplementary Materials

Effect of A- or B-Site Sc Doping on Sintering Temperature, Crystal Structure, Microstructure, and Properties of $BaZr_xTi_{1-x}O_3$ Ceramics

Kaituo Zhang [1], Tiantian Li [2] and Yuan Xu [3,*]

1 Henan Key Laboratory of Wire and Cable Structures and Materials, School of Cable Engineering, Henan Institute of Technology, Xinxiang 453003, China
2 Engineering Technology Education Center, Henan Institute of Technology, Xinxiang 453003, China;

3 College of Materials Sciences and Engineering, Henan Institute of Technology, Xinxiang 453003, China
* Correspondence: xuyuan94@163.com

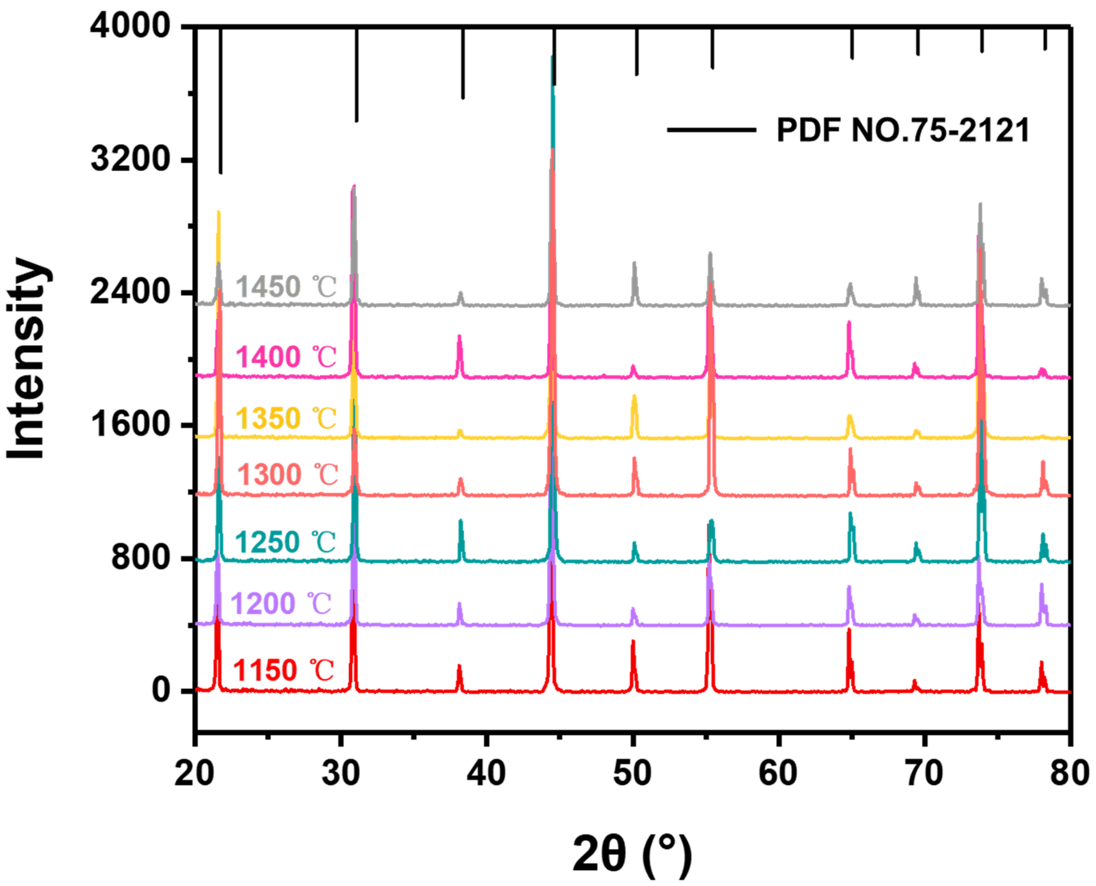

Figure S1. XRD patterns for the BZT-1.5mol % ceramics at different sintering temperatures.

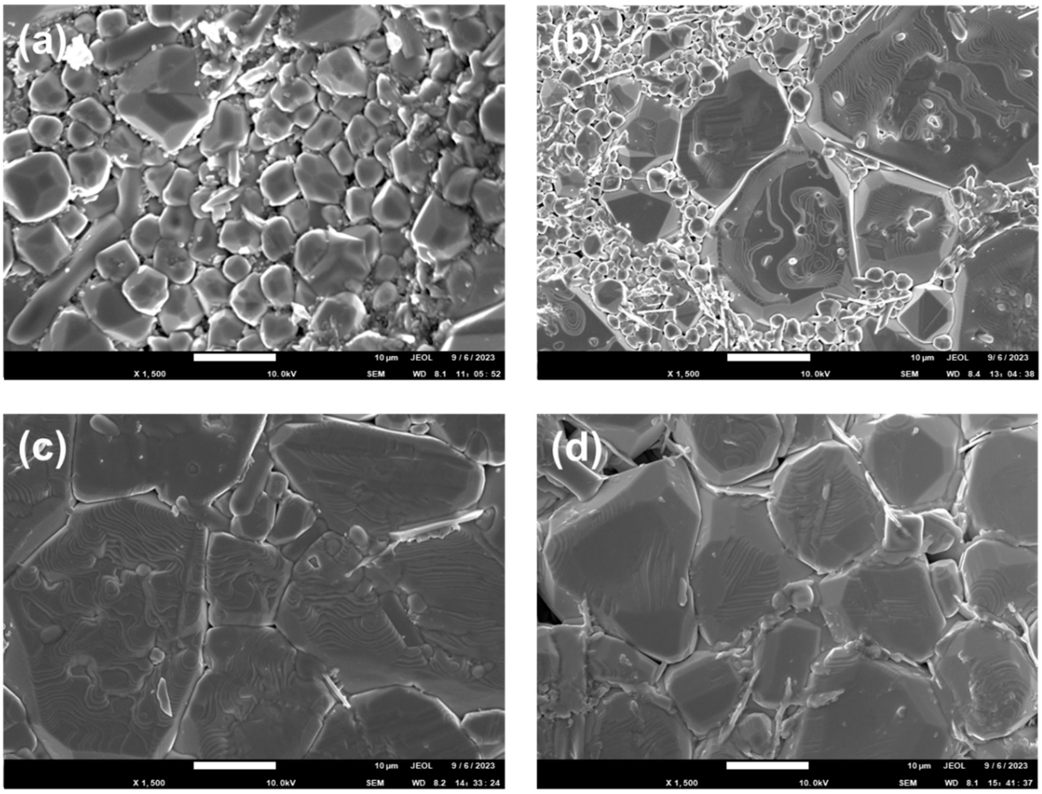

Figure S2. SEM images of BZT-1.5 % mol ceramics at sintering temperature of (a) 1150, (b) 1200, (c) 1250 and (d) 1350 °C.

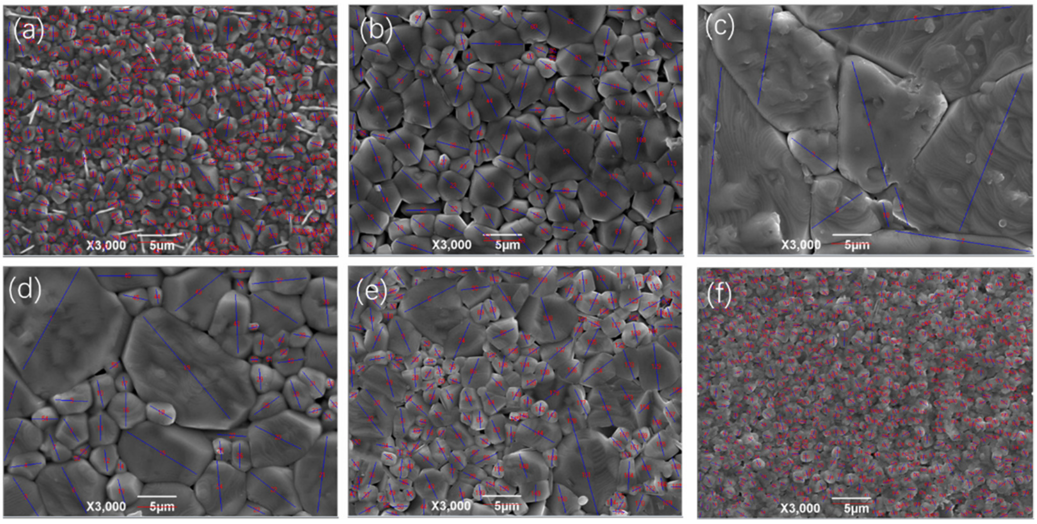

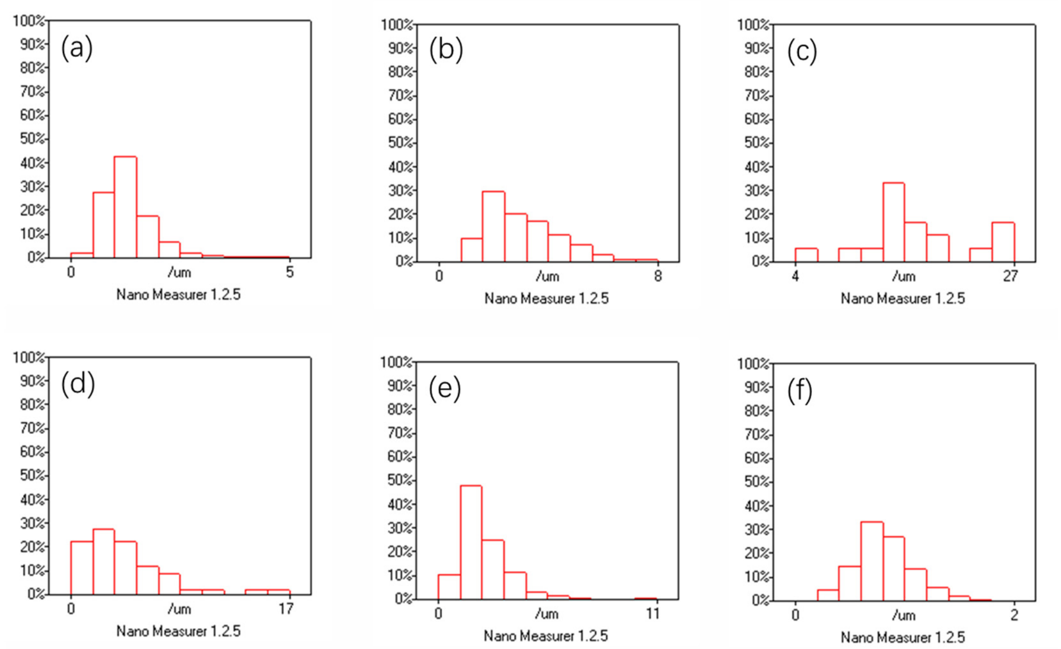

Figure S3. SEM images of BZT-x % mol ceramics (**a**) 0% mol, (**b**) 0.5% mol, (**c**) 1.0% mol, (**d**) 1.5 % mol, (**e**) 2.0% mol and (**f**) 2.5 mol and statistical chart.

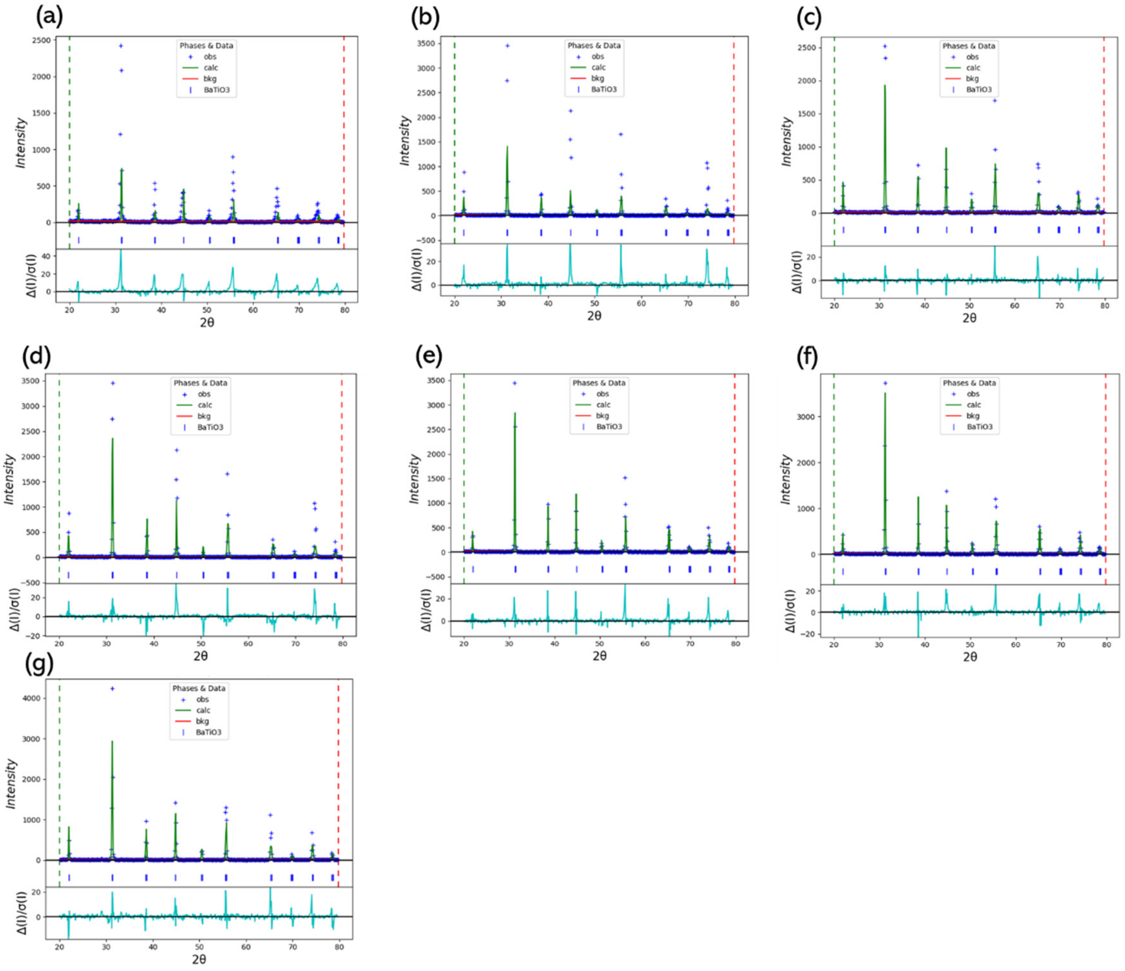

Figure S4. Rietveld refined room temperature XRD patterns for the BZT-x % mol ceramics. (**a**) 0% mol, (**b**) 0.25% mol, (**c**) 0.5% mol, (**d**) 1.0% mol, (**e**) 1.5 % mol, (**f**) 2.0% mol and (**g**) 2.5 mol.

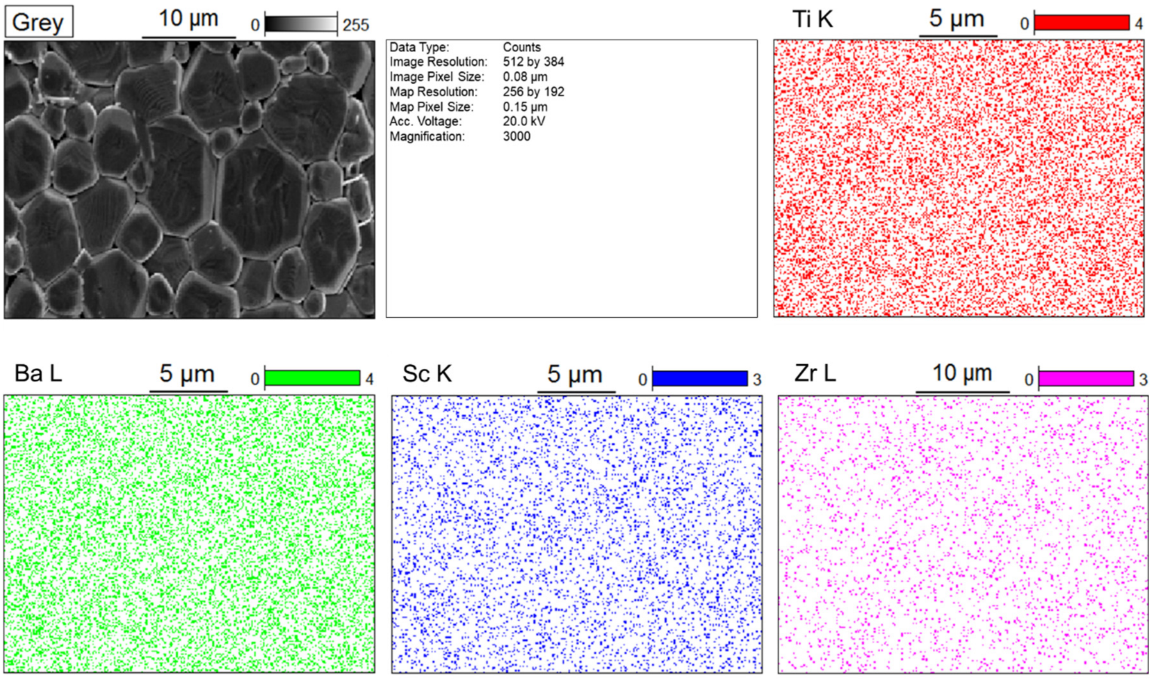

Figure S5. EDX map of 2.0% mol-BZT ceramics and correlation test parameter.

Table S1. Lattice constants fitting precision parameters of x-mol-BZT ceramics refined by Rietveld method.

Sc Content (%)	a = b	c	Reliability Factors		
			R_{wp}	R_p	χ^2
0 mol %	4.026819	4.078185	5.17%	4.26%	5.327
0.25 mol %	4.028764	4.083561	6.18%	4.15%	5.128
0.5 mol %	4.029351	4.096825	5.79%	3.78%	4.462
1.0 mol %	4.030215	4.091254	5.62%	3.53%	3.735
1.5 mol %	4.032451	4.083463	6.27%	3.57%	4.177
2.0 mol %	4.034568	4.076389	5.36%	3.43%	3.642
2.5 mol %	4.035672	4.071268	4.17%	3.17%	2.873

Table S2. The content of elements is determined by EDX test.

Element	Weight %	Weight Error (%)
O	17.41	+/- 0.51
Ti	19.06	+/- 0.82
Zr	6.27	+/- 0.43
Ba	56.41	+/- 1.61
Sc	0.85	+/- 0.35
Total	100.00	
